# Celluloid Handles for Instruments

**Published:** 1884-02

**Authors:** 


### CELLULOID HANDLES FOR INSTRUMENTS.

The cut of the instruments in Dr. St. Geo. Elliott's article gives but a very imperfect idea of their beauty. Nothing of the kind that we have ever seen approaches the tinted and mottled celluloid in appearance, and a set of pluggers or excavators finished after Dr. Elliott's plan is a thing of beauty.

